# *Mentha deleoi* (Lamiaceae): A New Species from Sicily

**DOI:** 10.3390/plants15040563

**Published:** 2026-02-11

**Authors:** Francesco Maria Raimondo

**Affiliations:** *PLANTA*/Center for Research, Documentation and Training, Via Serraglio Vecchio 28, 90123 Palermo, Italy; raimondo@centroplantapalermo.org

**Keywords:** Mediterranean flora, taxonomy, endemism, Sicily, biodiversity conservation, economic valorization

## Abstract

*Mentha deleoi* is described from Isola Grande, an islet in the Stagnone of Marsala (Trapani, NW Sicily), included within the homonymous Regional Oriented Nature Reserve. It is a perennial herb with an ephemeral annual cycle; in several morphological characters, it shows affinity with *M. pulegium*, a Eurimediterranean hemicryptophyte widely distributed in Sicily from coastal areas to high mountain habitats. Diagnostic and differential characters are provided, together with analyses of the biology, ecology, and chemistry of this new Sicilian endemic species. Owing to its extremely restricted distribution, small population size, and the potential impacts affecting the islet and its fragile habitat, resulting from centuries saliculture, a conservation plan is proposed, aimed at both in situ and ex situ protection.

## 1. Introduction

*Mentha* L. is one of the most complex genera of the *Lamiaceae* family, which reaches its greatest richness of genera and species in the Mediterranean area. In Europe and the Mediterranean, however, only a limited number of *Mentha* species are recognized, in contrast to the numerous infraspecific taxa—not always consistently accepted—and the many nothotaxa described. According to Harley [1], the European flora comprises eleven well-defined species, while the Mediterranean flora includes nine species [2]; in total, ten species are recognized in the flora of the European and Mediterranean area, as reported in the latest update of Euro+Med PlantBase [3]. Regarding the Italian flora, there are also nine specific taxa [4], four of which occur in Sicily [5].

In recent years, continued field observations of a small population of the genus *Mentha* have attracted particular interest. This population is well localized and confined to one of the small islands of the Stagnone di Marsala (Trapani, NW Sicily), namely Isola Grande, also known as “Isola Lunga” because of its narrow and elongated shape. This mint population—previously misidentified as *Mentha pulegium* var. *tomentella* (Hoffmanns. & Link) Cout. [6]—following a comparative chemical analysis with other Sicilian populations referred to the same taxon, has been shown to possess a different phytochemical profile, as well as a distinct ecology and biology.

It is a perennial plant with a very short vegetative cycle, growing on basic, calcareous, sandy–silty soils that, during part of the winter season, are subject to periodic stagnation of brackish water. Spatially and genetically isolated from other populations of the closely related *M. pulegium* L., it exhibits sufficient distinctive characters to be separated from them and treated as a distinct taxon. It is here described under the name *Mentha deleoi*, in memory of the chemist and botanist Prof. Antonino De Leo.

## 2. Results

### Taxonomic Analysis

**Figure 1:**
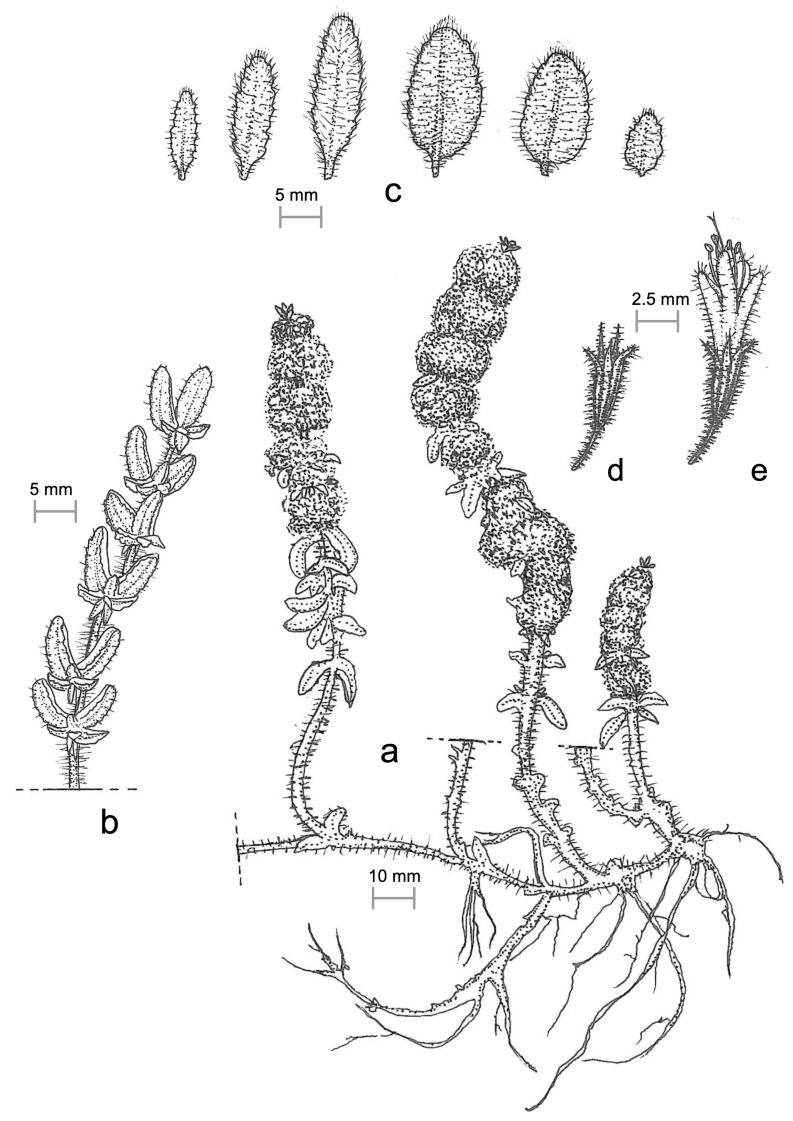
Iconographic drawing of the new species *Mentha deleoi* (drawing by Vincenzo Magro): (**a**) plant; (**b**) vegetative stem with opposite leaves and fascicles of axillary leaflets; (**c**) on the right basal leaves; on the left, leaves of the floral axis; (**d**) calix; (**e**) complete flower.

***Mentha deleoi*** Raimondo **sp. nov.** (Figure 1).

*Diagnosis*: Species herbacea perennis, ciclo effimero, omnibus organis aeriis dense villosa. Differt a *Mentha pulegium* L. foliis ovatis ad oblongis, plerumque convolutis; scapis floriferis erectis vel decumbentibus-ascendentibus, in plantis maiore magnitudine longioribus; verticillasteribus numerosis, densis, contiguis et globosis; floribus plerumque lilacinis, rarius albis.

**Figure 2:**
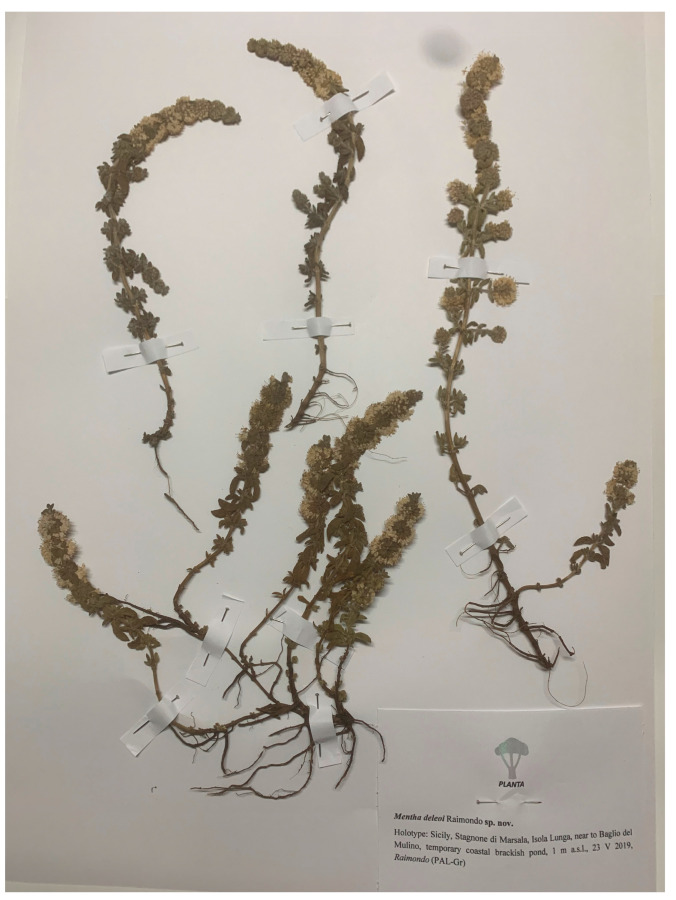
Holotype of *Mentha deleoi* preserved in PAL-Gr herbarium.

Holotype (Figure 2): Sicily, Stagnone di Marsala, Isola Grande, near Baglio del Mulino, in a temporary coastal brackish pond, 1 m a.s.l., 37°54′06″ N, 12°27′07″ E, 23.V.2019, Raimondo (PAL-Gr); Isotypes: PAL and FI.

**Figure 3:**
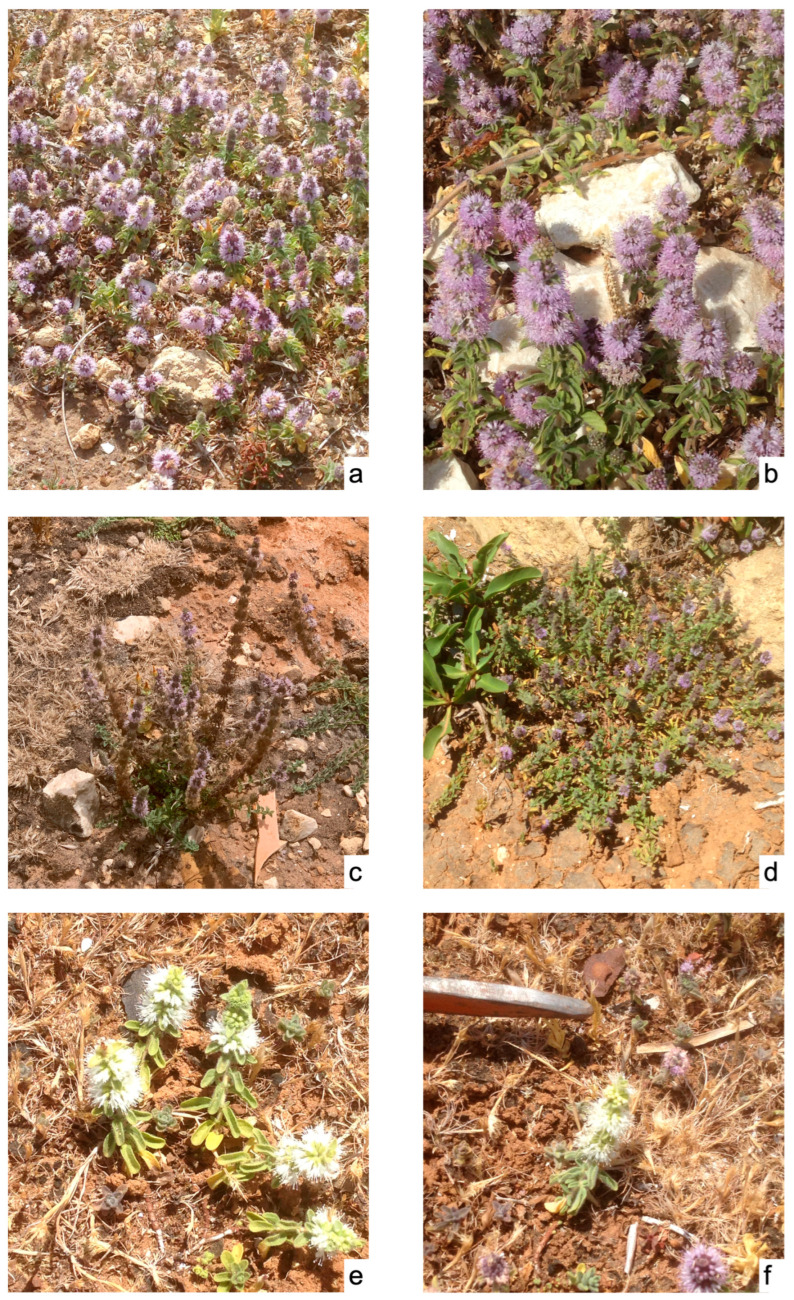
*Mentha deleoi* in flowering: (**a**–**c**) in the spring; (**d**,**e**) in the summer; (**e**,**f**) plants with white flowers.

*Description* (Figure 3): Perennial herbaceous plant with an ephemeral cycle, densely villous in all parts. Stems erect or plagiotropic, simple or slightly branched at the apex, 8–35 cm long, creeping on the ground up to over 30–35 cm. Cauline leaves densely glandular on the abaxial surface, shortly petiolate; blade ovate to oblong, 5–12 (–15) × 3–6 (–7) mm, flat to slightly convolute, apex obtuse to rounded, margin weakly dentate-crenate; axillary leaves much smaller, in fascicles of (3–) 5; bracts 3–9 × 2–3 mm. Flowers numerous in dense verticillasters, 10–12 mm in diameter, spaced 2–4 mm apart along plagiotropic-ascending axes. Calyx hispid, bilabiate, tubular, with 10 veins and 5 teeth: 2 longer and erect, 3 shorter and divergent. Corolla sparsely villous, bilabiate, generally lilac, though some individuals bear only white flowers (Figure 3e–f). Stamens 4, didynamous. Stigma bifid.

*Etymology:* The name of the new species commemorates Prof. Antonino De Leo (1905–1971), chemist and phytologist, and Professor of Systematic Botany at the University of Palermo. On 3 July 1971—just days after graduating in Agricultural Sciences from the University of Palermo—Prof. De Leo appointed the author as Curator of the Botanical Garden, introducing him to environmental and applied botany, which he pursued continuously for over fifty years. He was among the first researchers in Italy to initiate applied studies on the biology and chemistry of tropical and subtropical plants introduced and cultivated at the Botanical Garden, fields that he directed from 1968 until his untimely death in November 1971.

*Distribution and ecology*: A point-endemic species, to date known only from Isola Grande, Stagnone di Marsala (Trapani, NW Sicily). Thermo-xerophilous, growing on subsaline, clay–silty soils, partly stony; it is associated with *Limonium narbonense* Mill. and other therophytic species, including *Rumex bucephalophorus* L., *Blackstonia perfoliata* (L.) Huds., *Centaurium pulchellum* (Sw.) Hayek ex Hand-Mazz. Stadlm., Janch. & Faltis, *Polypogon subspathaceus* Req., *Convolvulus lineatus* L., *Desmazeria sicula* (Jacq.) Dumort, *Parapholis incurva* (L.) C.E.Hubb., *Scolymus grandiflorus* L., *Lysimachia arvensis* (L.) U.Manns & Anderb., *L. foemina* (Mill.) U.Manns & Anderb., *Limonium* sp.pl., *Bromus* sp., *Echium* sp., etc. The community has been attributed to the *Pulicario-Scirpetum savii* Brullo & Di Martino 1974 (*Isoetion* Br.-Bl. 1931, *Isoetalia* Br.-Bl. 1931), but today, especially due to the abundant presence of *M. deleoi* and *Limonium narbonense*, it deserves a different syntaxonomic interpretation.

**Figure 4:**
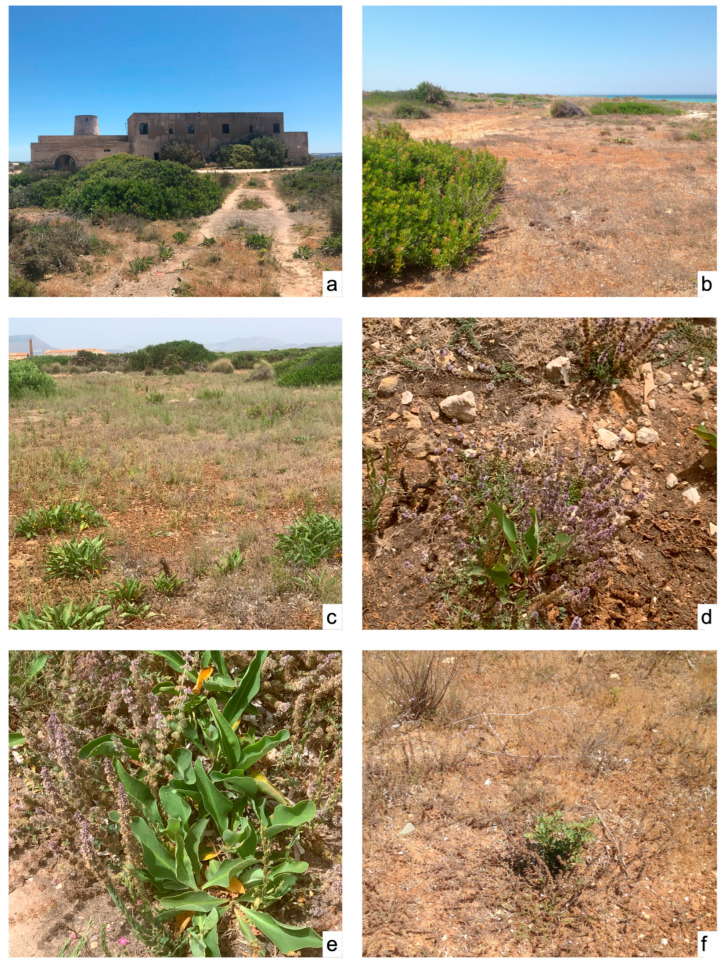
(**a**) Baglio del Mulino, a traditional building dedicated to salt-making activities near the area hosting the *M. deleoi* population; (**b**) coastal landscape in front of the area where the species is native: mint plants are highlighted at the end of their vegetative cycle; (**c**) general aspect of the phytocoenosis characterized by *M. deleoi*: note *Limonium narbonense* in full vegetative activity; (**d**,**e**) *M. deleoi* mixed with the thriving *Limonium narbonense*; (**f**) summer aspect of the *M. deleoi* colony at the end of fruiting and the vegetative cycle.

**Figure 5:**
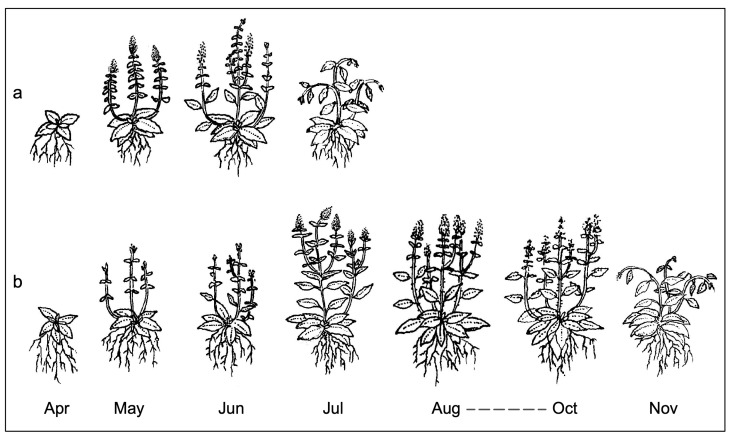
Scheme of the annual vegetative cycle of (**a**) *Mentha deleoi* and (**b**) *M. pulegium* in Sicily.

*Biology and phenology*: (Figure 4 and Figure 5) Hemicryptophyte rhizomatous with a short annual cycle, limited to the months of April–July; vegetative growth resumes in April; flowering from mid-May to late June; fruiting in June–July.

*Phytochemistry:* This plant is rich in secondary metabolites, several of which display notable biological activity. The main active compounds include isomenthone and pulegone [7].

*Conservation status:* According to the IUCN Red List Categories and Criteria [8], *Mentha deleoi* should be classified as Critically Endangered (CR B1ab (iii) + B2ab (iii)). The species is known from a single locality, with an estimated area of occupancy of approximately one hectare. Its habitat occurs adjacent to a service road used for saltworks operations, resulting in ongoing habitat degradation and a high risk of extinction.

*Conservation:* Based on the biological activities reported by Caputo et al. [7], which reveal the toxic effects of the main compounds of this species, its cultivation under controlled conditions replicating temporary moisture and salinity has been proposed as a sustainable approach for producing eco-friendly bioherbicides. This practice also serves as an *ex situ* conservation measure.

*Affinities:* This is a species closely related to *M. pulegium* (Sect. *Pulegium* (Miller) DC.), from which it differs mainly in its short biological cycle and in several morphological and ecological traits. It is distinguished by leaf morphology and indumentum, by the presence of axillary fascicles of small leaves, and by the density of the verticillasters along the flowering stem. Additional distinguishing characters include the pronounced asymmetry of the rather hirsute calyx, as well as the occurrence of individuals bearing only white flowers.

The main diagnostic morphological characters distinguishing *Mentha deleoi* from the closely similar *M. pulegium* are summarized in Table 1.

## 3. Discussion and Conclusions

In the past, in the flora of this insular region, apart from the numerous hybrid forms referred to the genus under study, several specific and infraspecific taxa were recognized, some of which are still regarded as taxonomically critical. According to Lojacono Pojero [9], the genus *Mentha* in Sicily was represented by numerous specific and infraspecific taxa, partly described by himself or by Gussone, as well as by several nothotaxa. Fiori [10], without recognizing their specific rank or taxonomic validity, mentioned almost all the species, varieties and hybrids described by Lojacono, relating them to the taxa accepted by him. Among Lojacono’s taxa, Fiori [10] and, more recently, Giardina et al. [11], also mention *Mentha lilibaea* and *M. syracusana*, which they include within *M. pulegium* L. Of the two, *Mentha lilibaea* Lojac. is reported from a locality near Marsala (Trapani), situated away from the coastal belt. Examination of the type material preserved at PAL excluded any affinity with the critical population from Isola Grande studied here; this population therefore remains previously undescribed, and its treatment as a new species is consequently justified.

**Figure 6:**
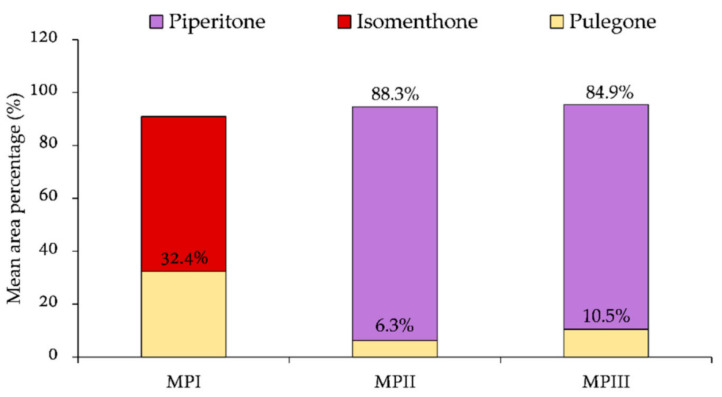
Chemotypes identified in *M. pulegium* plants collected in the three Sicilian sampling areas (I, II, and III). Percentage values reported in the graph bar refer to the average content of pulegone, isomenthone, and piperitone found in both EOs from leaves and flowers from each sampling point (from Ref. [7]).

As previously reported, phytochemical analyses highlighted the distinctiveness of the population from Isola Grande, located in the Stagnone of Marsala (Trapani), compared with other Sicilian populations from Castellana Sicula and Castronovo di Sicilia (both in the province of Palermo), attributed to *M. pulegium* s. str. [7]. The essential oil (EO) composition revealed clear and consistent chemotypic differences among the analyzed populations. In particular, the Isola Grande population (MPI) was characterized by the presence of isomenthone and the absence of piperitone, which was instead the predominant compound in the inland and hilly populations of Sicily (MPII and MPIII). Pulegone was the only compound shared by all populations and occurred at relatively higher levels in the coastal population (Figure 6). These distinctive chemical profiles were interpreted as an adaptive response to the specific pedoclimatic conditions of the habitat, including soil salinity [12].

Based on continuous field observations of the biological cycle and diagnostic morphological traits, the taxonomic interpretation has been revised, supporting the recognition of the Isola Grande population as a distinct new species. As the species is at risk, protecting its habitat is a priority.

In conclusion, the proposal by the company “Isola Longa”—the owner of the area—to implement “active conservation” measures for *M. deleoi* is noteworthy. These measures involve cultivating the species on the island itself, making use of abandoned structures along the coastline, particularly concrete tanks formerly used for fish farming.

In this context, revising the regional framework governing protected areas becomes a priority, with the introduction of a new category of nature reserve corresponding to the Spanish model of a “micro-reserve”, characterized by specific management regulations and implementable by private landowners under the supervision of public authorities [13].

## 4. Materials and Methods

**Figure 7:**
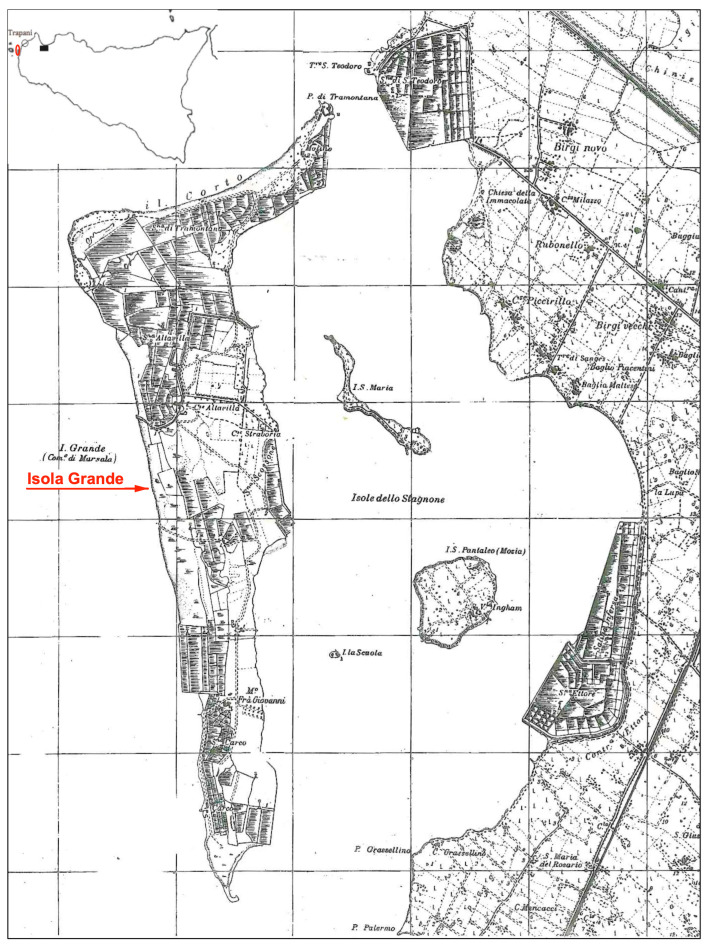
Location of Isola Grande within the Stagnone of Marsala (Trapani), NW Sicily.

This study was conducted through periodic field observations of the population in its natural habitat on Isola Grande, followed by explorations on the small islets of the Stagnone of Marsala and along the nearby Sicilian coast, from Marsala to Trapani (Figure 7). Dried study materials were subsequently analyzed and taxonomically compared with herbarium specimens from various Mediterranean origins, preserved at several herbaria available online.

For the comparison of morphological characters between *M. deleoi* and *M. pulegium*, specimens from an inland, hilly population collected in Castellana Sicula (PA), Sicily, were used. All data are reported in Table 1, with micromorphological information on calyx trichome density and glandular trichome size [7].

Relevant references included studies on the flora and vegetation of the small islet hosting the critical population [6,14], as well as recent phytochemical and biological activity investigations conducted on several Sicilian populations of *M. pulegium*, including the one studied here [7,12].

The nomenclature of taxa cited in the text follows the International Plant Names Index [15], while that of syntaxa follows the Prodromo della vegetazione d’Italia [16].

## Figures and Tables

**Table 1:** Morphological comparison between *Mentha deleoi* and *Mentha pulegium* from Sicily.

Character/Taxon	*Mentha deleoi*	*Mentha pulegium*
Stems	8–35 cm long, simple or slightly branched at the apex	15–45 cm long, distinctly branched
Leaves	Cauline leaves shortly petiolate; blade ovate–oblong, 5–12 (–15) × 3–6 (–7) mm, apex obtuse–rounded, margin weakly dentate–crenate; axillary leaves smaller, in fascicles of (3–) 5	Cauline leaves petiolate; blade ovate–lanceolate, 20–30 × 4–15 mm, apex acute, margin serrulate; axillary leaves smaller, generally in pairs
Bracts	3–9 × 2–3 mm	3–12 × 2–5 mm
Inflorescence	Dense verticillasters, 10–12 mm in diameter, spaced 2–4 mm apart along the stem	Verticillasters, 6–8 mm in diameter, spaced 6–12 mm apart along the stem
Calyx teeth	5, 2 longer and erect, 3 shorter and divergent	5, subequal, erect
Trichomes	Calyx trichome density (13.7 ± 2.8 n mm^−2^); leaf glandular trichome head diameter 100.1 ± 3.7 μm	Calyx trichome density (19.0 ± 1.6 n mm^−2^); leaf glandular trichome head diameter 104.5 ± 10.2 μm

## Data Availability

The original contributions presented in this study are included in the article. Further inquiries can be directed to the corresponding author.
